# Effects of Bioadvection by *Arenicola marina* on Microphytobenthos in Permeable Sediments

**DOI:** 10.1371/journal.pone.0134236

**Published:** 2015-07-31

**Authors:** Arjun Chennu, Nils Volkenborn, Dirk de Beer, David S. Wethey, Sarah A. Woodin, Lubos Polerecky

**Affiliations:** 1 Max Planck Institute for Marine Microbiology, Bremen, Germany; 2 Department of Biological Sciences, University of South Carolina, Columbia, United States of America; Auckland University of Technology, NEW ZEALAND

## Abstract

We used hyperspectral imaging to study short-term effects of bioturbation by lugworms (*Arenicola marina*) on the surficial biomass of microphytobenthos (MPB) in permeable marine sediments. Within days to weeks after the addition of a lugworm to a homogenized and recomposed sediment, the average surficial MPB biomass and its spatial heterogeneity were, respectively, 150-250% and 280% higher than in sediments without lugworms. The surficial sediment area impacted by a single medium-sized lugworm (~4 g wet weight) over this time-scale was at least 340 cm^2^. While sediment reworking was the primary cause of the increased spatial heterogeneity, experiments with lugworm-mimics together with modeling showed that bioadvective porewater transport from depth to the sediment surface, as induced by the lugworm ventilating its burrow, was the main cause of the increased surficial MPB biomass. Although direct measurements of nutrient fluxes are lacking, our present data show that enhanced advective supply of nutrients from deeper sediment layers induced by faunal ventilation is an important mechanism that fuels high primary productivity at the surface of permeable sediments even though these systems are generally characterized by low standing stocks of nutrients and organic material.

## Introduction

Benthic phototrophs such as diatoms, cyanobacteria, chlorophytes and dinoflagellates, which are commonly referred to as microphytobenthos (MPB), are a major contributor to the primary productivity [1, 2], food web [3] and sediment stability [4] in coastal ecosystems. Their activity and distribution are mainly determined by light climate, nutrient levels and grazing pressure, and exhibit great spatio-temporal variability from mesoscopic to regional [5, 6] and daily to seasonal scales [7, 8]. In this study we focus on the effect of nutrient supply and grazing on the milli-to-centi-meter scale distribution of surficial MPB.

MPB cells at the sediment surface rely on transport of nutrients to the sediment–water interface either from the overlying water column or from the underlying porewater, where nutrient concentrations are typically orders of magnitude greater than in the overlying water [9]. In fine silt or muddy sediments, nutrient transport from depth to the surface is diffusive, and therefore typically slower than in permeable sandy sediments where transport can additionally occur by porewater advection [10]. Advective transport in the upper layers of permeable sediment is driven by water column hydrodynamics as well as by ventilation by benthic infauna [11–13]. In fact, the biologically driven advection, termed bioadvection, can generate porewater flows much deeper in the sediment than those driven by water column hydrodynamics [14, 15]. As a consequence, ventilation by benthic infauna is thought to be a significant driver of nutrient transport to MPB at the sediment surface [16, 17].

In intertidal sediments, e.g., in the Wadden Sea, the lugworm *Arenicola marina* is a classic ecosystem engineer that bioturbates, i.e. irrigates and reworks, immense volumes of sediment [18, 19]. Lugworms live head-down in 20–40 cm deep J-shaped blind-ending burrows and feed on fresh detrital material that is transported to depth at the blind-end of the burrow through their ventilation and feeding activities (reviewed by [20, 21]). Lugworms ventilate their burrows through peristalsis, which leads to enhanced transport of oxygen and other solutes from the overlying water into the sediment [22]. The requirement to ventilate the blind-ending burrow restricts lugworms to permeable sandy sediments [14].

Sandy sediments typically have very low organic content, and the fact that lugworm populations thrive in such sediments has elicited considerable discussion on their food supply [21, 23]. However, it should be noted that small pools can have a high turnover rate [24]. Indeed, Hylleberg [25] introduced the concept of “gardening” as the process of stimulating growth of microorganisms in the burrow through oxygen supply and their subsequent use as food. However, stable isotope analysis suggests that MPB is the major component of the lugworms' diet, and that the abundance of macrobenthic organisms generally depends upon the MPB productivity [26]. The idea that lugworms might additionally maintain their "garden" also at the sediment surface has hitherto not been investigated.

In this study, we aimed to explore this idea by elucidating the coupling between the bioturbation activities of lugworms and the growth of MPB at the sediment surface. We hypothesized that the bioadvected porewater transport induced through burrow ventilation by a lugworm leads to an increase in the surficial MPB biomass, whereas the reworking activities, including sediment ingestion and defecation, leads to depletion and burial of surficial MPB. Our goal was to understand the combined effect of the opposing forces of porewater bioadvection and sediment reworking. To do so, we used high-resolution hyperspectral imaging to quantify the surficial MPB biomass in three experimental settings: 1) laboratory experimental containers with and without lugworms to quantify the combined effects of bioadvection and sediment reworking; 2) laboratory experimental containers with and without mimics of lugworm irrigation to study the effect of bioadvection in isolation from all other faunal effects and 3) in-situ experimental plots with and without lugworms where the MPB growth occurred under natural conditions, but was potentially affected by factors other than the presence or absence of lugworms, such as grazing by other benthic animals and physical disturbance by waves or currents. Additionally, we performed numerical model simulations to evaluate whether the spatial patterns of bioadvection-induced nutrient flux at the sediment surface would conform to the patterns of MPB distributions observed in the experiments.

## Methods

### Sediment sampling

Experiments were performed with sandy sediments collected from intertidal sand-flats at two locations: Oyster Landing, Winyah Bay, South Carolina, USA (33.35°N, 79.19°W) and Koenigshafen, Sylt, Germany (55.0252°N, 8.436°E). No permit is required for collecting sediment at the Oyster Landing site. Sediment sampling and field experiments at the Koenigshafen site were done under the general agreement that exists between the Nationalpark Schleswig-Holsteinisches Wattenmeer and the Wadden Sea Station Sylt of the Alfred Wegener Institute for Polar and Marine Research, which allows participating scientists to take samples and perform field experiments. No endangered or protected species were collected for this research.

For all experiments, sediments from the deeper reduced layer and the surficial oxidized layer were sampled and processed separately. The transition depth between the two layers was chosen through visibly different coloration. Sediments were sieved through a coarse mesh (5 mm) to exclude large infauna and other objects, homogenized and recomposed in a similar way as the original oxidized and reduced layers. They were subsequently left to settle for at least 24 hours before the commencement of experiments.

### Hyperspectral imaging of MPB biomass

MPB biomass in surficial sediments was quantified using the hyperspectral imaging system *hypersub* and experimental protocols described by Chennu et al. [6]. Briefly, the system captures back-scattered light from the sediment and, using a spectral reference, converts the detected signal into reflectance spectra (wavelength range 400–900 nm, spectral resolution about 2 nm). These spectra are used to calculate a microphytobenthos index (MPBI) at each location in the spectral image, from which chl *a* concentrations in the top millimeter of the sediment are estimated by using a linear calibration [6]. The non-destructive character of the imaging method allows monitoring of the spatial patterns of chl *a* concentrations over the same sediment region with high spatial and temporal resolutions.

During this study, the *hypersub* system was positioned 0.8–1.0 m above the sediment surface and the scanning parameters were adjusted to obtain hyperspectral images with a spatial resolution of 1 × 1 mm per pixel. In-situ measurements were performed under ambient sunlight, whereas measurements in experimental tanks (see below) used partially shaded ambient light together with a supplemental illumination from overhead halogen lamps. Measurements conducted during the night involved illumination of the sediment surface only by the halogen lamps. The illumination was restricted to the duration of the scan. Since each scan took about 10 min and subsequent scans were separated by at least 1 hour, we assume that the effects of the artificial illumination on vertical migration of MPB within the surficial sediment layer were negligible [6]. In all measurements, a gray plastic board with a matte surface finish was used as a spectrally flat reference.

MPB biomass was quantified as weight of chlorophyll *a* per volume of porewater (μg chl *a* mL^-1^ porewater) in the top millimeter, and was calculated in each pixel of the scans from the measured MPBI as chl *a = S ×* (*MPBI-MPBI*_*0*_), where *S* = 1776 μg mL^-1^ PW and *MPBI*_*0*_ = 0.030. These calibration values correspond to the measured grain-size of the studied sediment (125–250 μm), which was assumed not to vary significantly over the scanned sediment regions [6]. The volumetric units of surficial chlorophyll can be converted to areal units [6] but this was not done in this study for simplicity. In addition to chl *a* maps, which are presented here as false-color images, true-color images of the scanned sediment regions were generated by using reflectance values at specific wavelengths as intensities of the red (640 nm), green (550 nm) and blue (460 nm) channels in composite RGB images.

### Laboratory experiment in the presence and absence of lugworms

To study the effect of lugworm activity on the surficial MPB biomass under controlled conditions, incubation experiments were conducted in a greenhouse laboratory at the Wadden Sea Station Sylt (Germany) in summer 2010. Six containers (area 18.5×18.5 cm, 20 cm height) were filled with recomposed permeable sediment from the Koenigshafen site and submerged in a large tank with a continuous input of fresh seawater at 18°C with a salinity of 31–32 on the practical salinity scale. After one day, single lugworms (wet weight 4.33 ± 0.4 g) were added to three of the six containers, corresponding to a density of 30 ind m^-2^ which is typical of the collection site [27]. The remaining three containers were used as controls. The sediment surface was exposed to natural illumination (incident PAR ~285 μmol photons m^-2^ s^-1^) shaded by the roof of the greenhouse. Hyperspectral scans of the sediment surfaces were made 1, 4 and 11 days after the lugworms were added.

### Laboratory experiment in the presence and absence of a lugworm-mimic

To study the effect of burrow ventilation by a lugworm in isolation from its sediment reworking activity, similar incubation experiments as described above were conducted using a mechanical lugworm-mimic instead of real lugworms. This was done in an open-air laboratory at the Baruch Marine Field Laboratory of the University of South Carolina, USA in summer 2011. Six cylindrical containers (diameter 15 cm, height 18.5 cm) were filled with recomposed permeable sediment from the Oyster Landing site. The lugworm-mimic was administered through the use of the “robolug” system [28], which allows realistic imitation of porewater advection produced by lugworms. It consisted of a thin (1.6 mm inner diameter) tube, with one end connected to a peristaltic pump and the other end entering the sediment containers from the side and buried (14 cm deep) in the sediment at the central axis of the container. The use of pulsed unidirectional pumping that delivered 0.25 mL pulses of seawater at a frequency of 6 pulses per minute through the tube outlet (2.5 mm diameter) ensured that the average pumping rate (1.5 mL min^-1^) as well as the source pressures resembled those induced by real “lugworm pumps” [15, 21, 28]. Additionally, to account for the fact that the oxygen concentration in the water entering the sediment from the lugworm's injection pocket is reduced due to lugworm's respiration [13, 29], the water pumped by the lugworm-mimic was maintained at approximately 30% air saturation by bubbling with N_2_ gas. The robolug outlets were set up in three replicate containers while three containers with no active porewater flow within the sediment were used as controls. All six containers were incubated in a large tank with continuous input of fresh seawater at 25°C with PSS salinity between 30–32. Sediment surface was exposed to natural illumination (PAR 90–150 μmol photons m^-2^ s^-1^) shaded by the roof of the open-air laboratory. Hyperspectral scans of the sediment surfaces were made in about one hour intervals over a period of four days using halogen lamp illumination as described above.

### Modeling of nutrient flux in the containers with a lugworm-mimic

Based on the different spatial profiles of MPB growth observed in the containers of the lugworm-mimic experiment, we hypothesized that under diffusive conditions the MPB growth at the sediment–water interface was limited by nutrient supply from the underlying sediment, while this limitation was lifted via advective transport induced by the lugworm-mimic. To test this we modeled the distribution of the flux of the growth-limiting nutrient at the sediment–water interface using the Comsol Multiphysics software (v4.3a from www.comsol.com). The no-mimic containers were modeled with the “Transport of dilute species” module and the robolug containers with the “Reacting flow in porous media” module of the software. Both simulations were done in 3D as a temporal evolution from an initial state towards an eventual steady state.

The modeled geometry consisted of two sub-domains: a porous medium with the same porosity (0.39), permeability (2.95 × 10^−12^ m^2^) and dimensions (see above) as the sediment in the experimental containers, and a thin layer of water above. The latter domain was introduced to be able to fix the nutrient concentration at some distance above the sediment–water interface to that in the overlying water (see below). For the situation with no porewater flow, this distance corresponds to the thickness of the diffusive boundary layer (DBL), which ranges between 0.1 mm and 1 mm depending on the velocity of the laminar flow above the sediment–water interface [30]. Therefore, the thickness of the thin water layer in our model was set to 0.5 mm in both modeled scenarios (with and without porewater flow). This numerical choice does affect the absolute values of the calculated nutrient fluxes but not their spatial patterns at the sediment–water interface.

The robolug outlet was approximated by a sphere with a radius of 2.5 mm, which was chosen to simplify numerical simulations. The geometrical arrangement of the outlet at a large distance (14 cm) from the sediment–water interface was similar to that employed in the “pocket injection” model of Meysman et al. [14, 31], where it was demonstrated that the hydraulic forces exerted by ventilating lugworms in permeable sediments can be adequately abstracted as emanating from a sphere located at the depth of the feeding pocket. Water injection through the robolug outlet at the experimental value of 1.5 mL min^-1^ was achieved by setting a constant flow-velocity of 0.816 mm s^-1^ across the surface of the injection sphere, which accounted for the porosity of the medium. Boundary conditions for the porewater flow were set to zero-flow (i.e., no slip) at the outer and lower boundaries of the domain (corresponding to the container walls) and to zero pressure at the upper domain boundary (corresponding to the top of the water layer).

The initial nutrient concentration in the sediment sub-domain was set to zero, which represents the porewater replacement by the initial sediment processing (homogenization and recomposition). Since the ambient seawater overlying the containers in the experimental tank had negligible nutrient concentrations and was well-mixed, the nutrient concentration in the injected water (when modeling the robolug containers) as well as at the top of the thin water layer above the sediment–water interface was set to zero. Nutrient generation was assumed to occur at a constant rate (1 μmol m^-3^ s^-1^) throughout the entire sediment sub-domain. These assumptions were adequate since the modeled nutrient flux scale proportionally to this rate, and the aim of the model was to obtain their relative spatial distribution and not their absolute values. The identity of the nutrient was not important in the model as long as its concentration could become limiting for MPB growth under diffusive conditions.

### In-situ experiment in the presence and absence of lugworms

To study the effect of lugworm activity on MPB distributions under natural conditions, experimental plots with and without lugworms were established in the Koenigshafen site in summer 2010. The site contained abundant lugworms and was close to the area investigated previously by Volkenborn et al. [22]. Replicate plots were established by burying open-top mesh bags (diameter 18 cm, 25 cm deep, mesh size 1 mm) into the sediment and surrounding them with a horizontal exclusion mesh (50 × 50 cm, mesh size 1 mm) placed at a depth of 10 cm. The mesh bags contained recomposed sediment from the site (permeable sandy sediment, grain-size 125–250 μm and porosity 0.39). Three days after the establishment of the plots, 4 small lugworms (lugworm wet weight: 1.46 ± 0.30 g; total length: 7.5 ± 0.8 cm) were added to each of three of the six mesh bags. This corresponds to a lugworm abundance of about 150 individuals m^-2^, which is within the range of small-sized lugworm densities in this region [27]. Hyperspectral scans of the plots were made 5 weeks after the introduction of the lugworms into the open-top mesh bags and after a series of calm weather days without evident signs of sediment resuspension. Due to technical complications, scans were performed successfully only in two out of three replicate plots with lugworms. Occasional counts of fecal mounds within the plots and the collection of worms at the end of the experiment (8 weeks) confirmed that all transplanted lugworms remained active over the course of the experiment.

## Results

### MPB distribution in laboratory containers with and without lugworms

**Fig 1:**
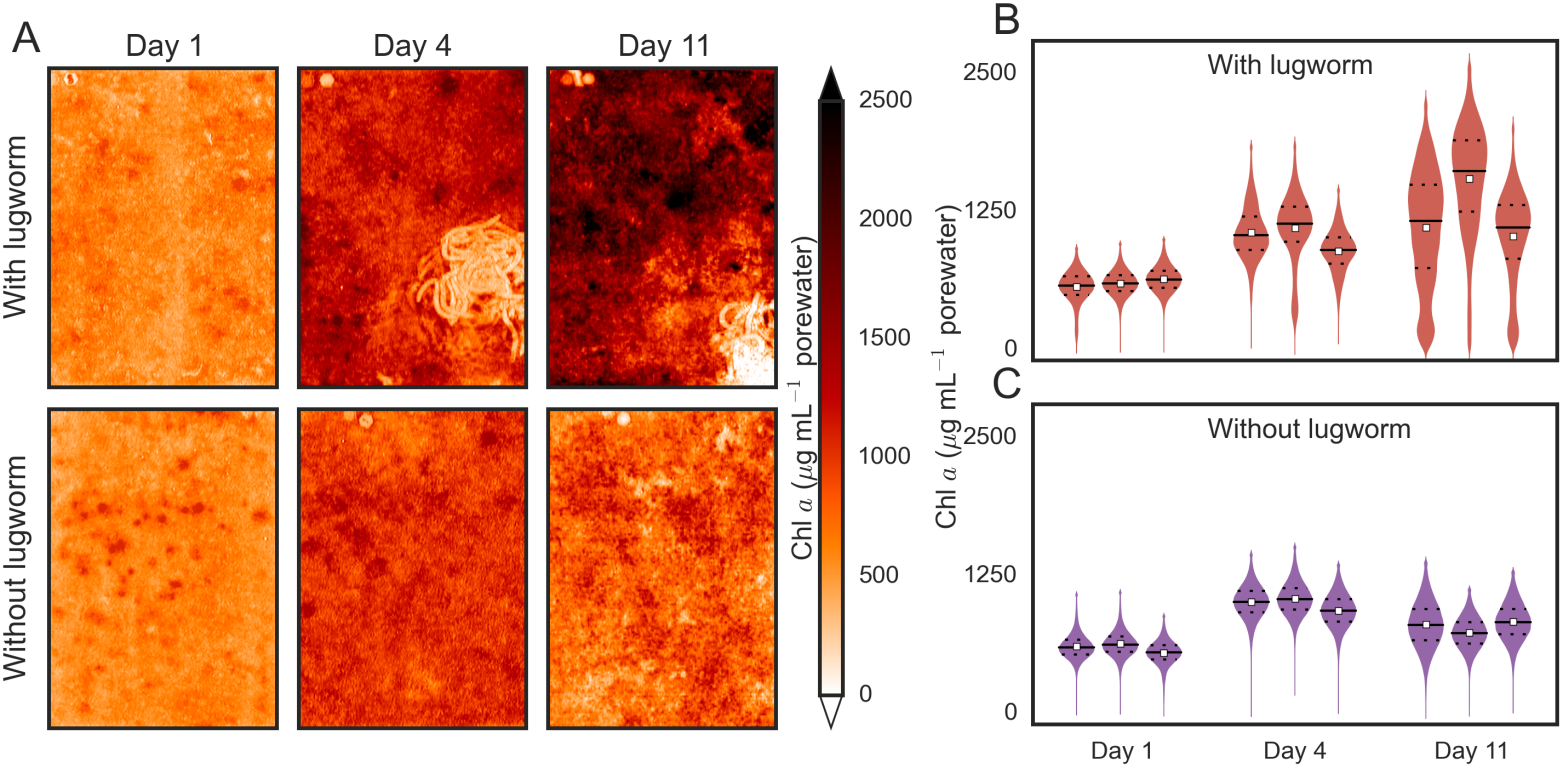
Chlorophyll *a* maps at the surface of permeable intertidal sediments in experimental containers with and without lugworms. Panel A shows examples from the same containers taken at the beginning, after 4 days and after 11 days of incubation. Panels B and C show the corresponding violin plots for the surficial chl *a* concentrations derived from three replicate containers, where the inner horizontal lines indicate the quartile levels and the square markers the average values.

Chlorophyll *a* maps obtained in the laboratory containers showed that the surficial MPB biomass overall increased during the course of incubation but the variation generally entailed both a decrease and an increase (Fig 1). In the treatment with a lugworm, patches where chl *a* concentrations decreased and increased were spatially contiguous and corresponded, respectively, to the fecal mounds and sediment regions unaffected by reworking (Fig 1A, top row). In contrast, patches where MPB biomass decreased and increased in sediments without a lugworm were smaller and randomly distributed across the sediment surface (Fig 1A, bottom row).

By analyzing the chl *a* concentrations averaged over the entire sediment surface, we found that the surficial MPB biomass in containers with and without lugworms increased similarly during the first 4 days, but the increase was significantly greater in the lugworm containers after 11 days of incubation (S1 Table). The similar increase in both treatments during the first four days was possibly a result of an increased nutrient release from the initial homogenization of the sediment (see Discussion).

**Fig 2:**
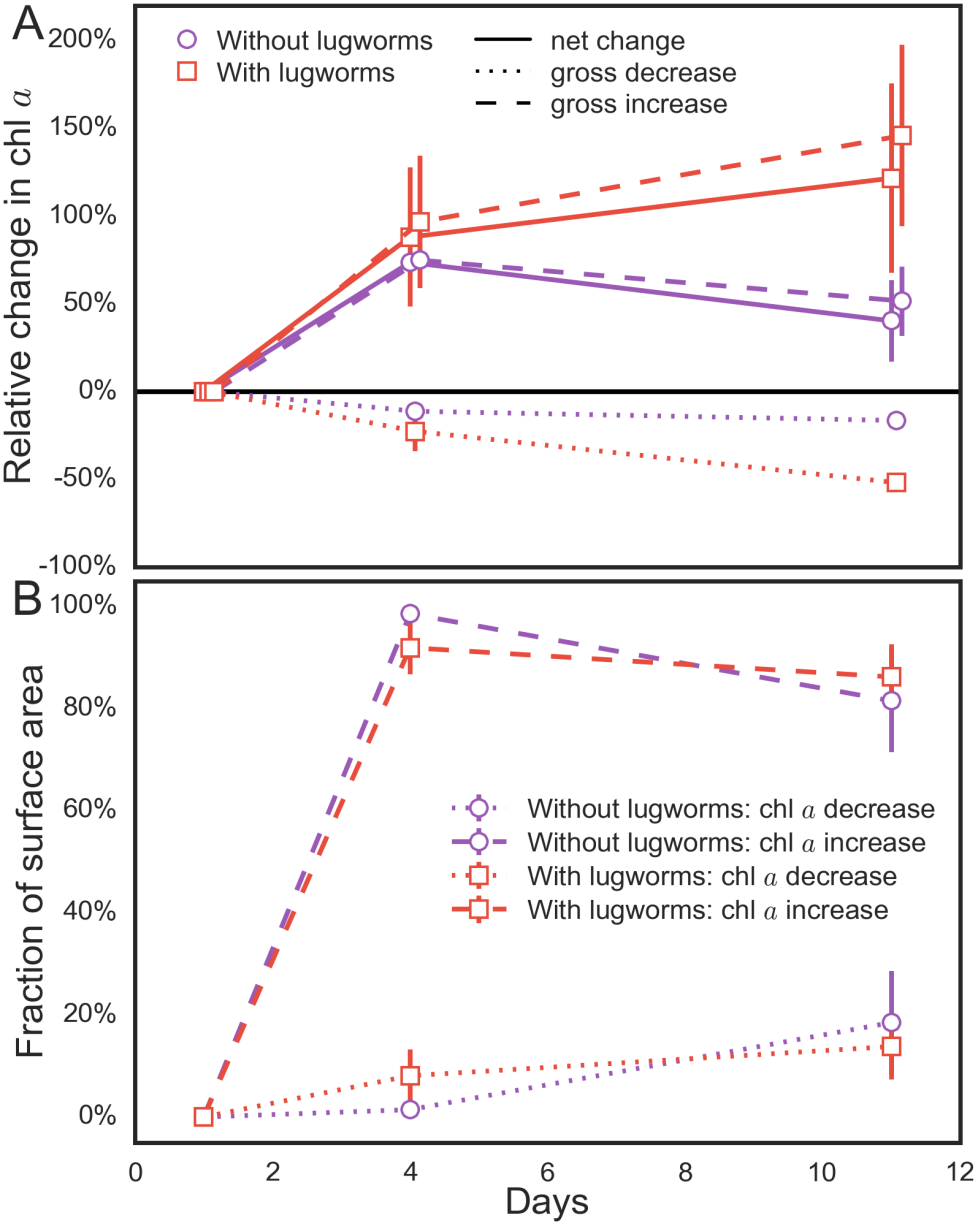
The dynamics of the surficial chlorophyll *a* concentration in the treatments with and without lugworms. Panel (A) shows the averages of three replicate containers described in Fig 1. The net relative change (solid line) in chl *a* shown in the top panel consisted of a gross increase (dashed lines) and a gross decrease (dotted lines) distributed over the fraction of the sediment surface area shown in panel (B).

To better highlight the effects of a lugworm on the surficial MPB biomass, we additionally analyzed the sediment areas where the chl *a* concentrations increased and decreased separately. We found that, for example, after 11 days of incubation the net 120% increase in the average surficial chl *a* concentrations comprised a 145% gross increase in areas unaffected by reworking and a 52% gross decrease in the defecated sediment (Fig 2A). The latter value indicates that lugworms digested approximately half of the pigmented biomass in the ingested sediment. A similar comparison in the lugworm-free containers revealed that the corresponding 40% net increase after 11 days of incubation comprised only a 52% gross increase and a 16% gross decrease (Fig 2A). Thus, in addition to significantly affecting the overall surficial MPB biomass, lugworm activity induced also a significant increase in its spatial heterogeneity (S1 Table). Interestingly, the cumulative areas where the gross increase and decrease occurred were similar for both treatments (e.g., 80–86% and 14–20% of the total sediment surface, respectively, after 11 days of incubation; Fig 2B).

### MPB distribution in the presence and absence of a lugworm-mimic

**Fig 3:**
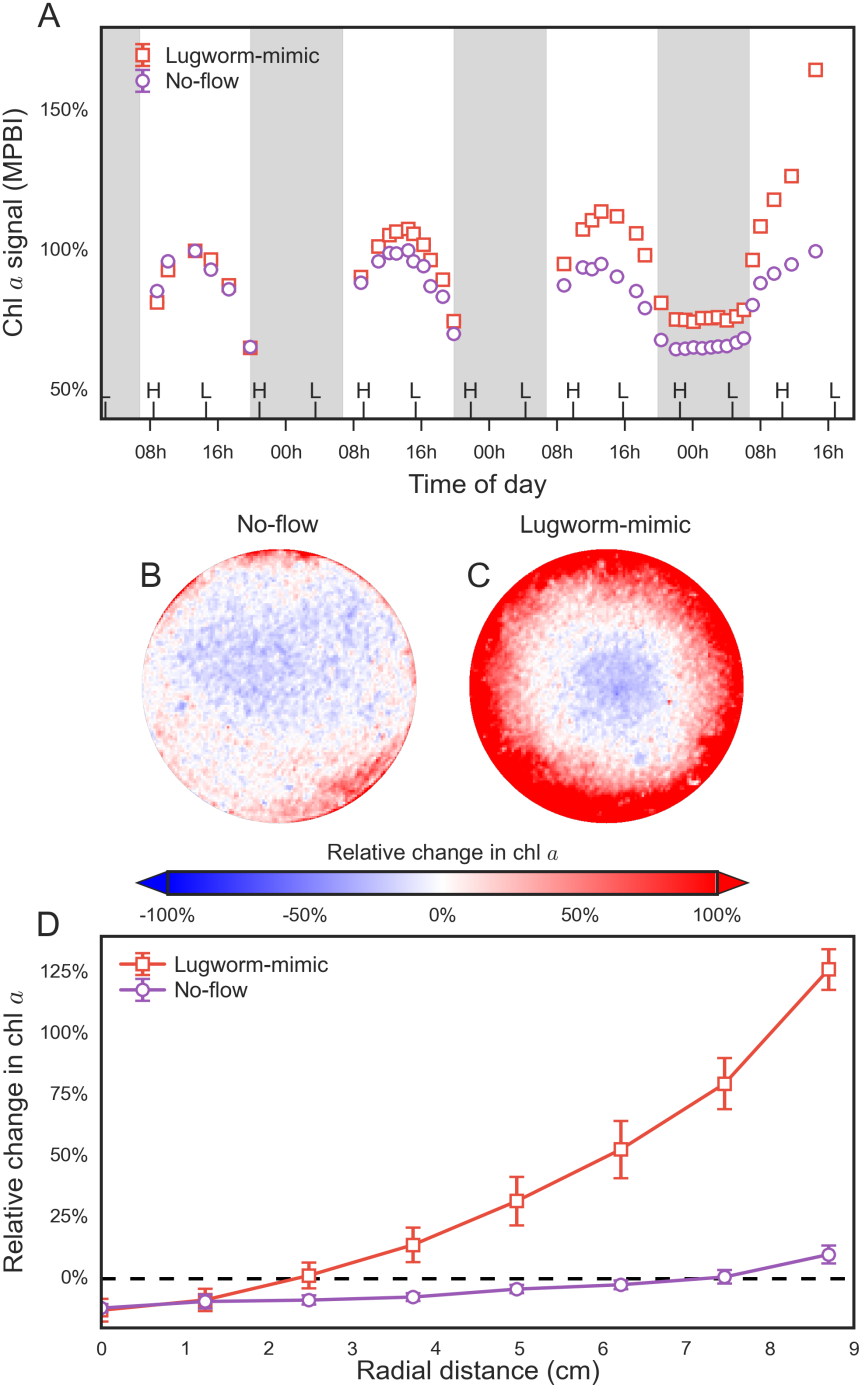
Dynamics of chlorophyll *a* concentrations at the surface of permeable sediments incubated in containers with and without a lugworm mimic. (A) The mean and standard deviation of the MPBI values averaged over the entire sediment surface in the containers, as derived from three replicates for each treatment. Error bars are smaller than the symbols except for the last data-point. Shaded and non-shaded parts indicate night and day time respectively. (B-D) Relative changes in the surficial chl *a* concentrations between the first and last day of the incubation, shown as false-color images (B, C) and as radial profiles (D) averaged over annular sections derived from 3 replicate measurements for each treatment.

To obtain a simplified view of the temporal variability of the surficial MPB biomass in the experimental containers with and without the lugworm-mimic, the spectral index used for the quantification of MPB biomass (MPBI) was averaged over the entire sediment surface for each replicate container (Fig 3A). In general, the MPBI signal for both types of containers exhibited diel oscillations, with maxima reached each day around noon and minima lasting during most of the night. This MPBI dynamic was due to the vertical migration of MPB [6].

As argued by Chennu et al. [6], the maximal values of the MPBI measured each day correspond to the maximal concentrations of the light-exposed MPB at the sediment surface for that day. Over the 4-day incubation, our data showed that the daily MPBI maxima (averaged over the entire sediment surface) remained approximately constant in the no-mimic containers but increased to about 160% in the containers with a lugworm-mimic (Fig 3A). This means that within 4 days the MPB community responded to the enhanced upward porewater transport by enhanced growth, while there was no significant net growth in the no-mimic treatment.

Using the daily MPBI maxima, we calculated for each pixel the relative change in the surficial MPB biomass between the first and fourth day of incubation (examples shown in Fig 3B and 3C). Additionally, we averaged these relative changes over annuli of increasing radius and plotted the averages as a function of the radial distance from the center of the container (Fig 3D). The results revealed that in the lugworm-mimic treatment the MPB biomass was progressively higher towards the edges of the containers, while it was slightly decreased in the central region of the containers. In contrast, the no-mimic containers showed no such spatially distinct patterns.

### Modeling of nutrient fluxes in the presence and absence of a lugworm-mimic

**Fig 4:**
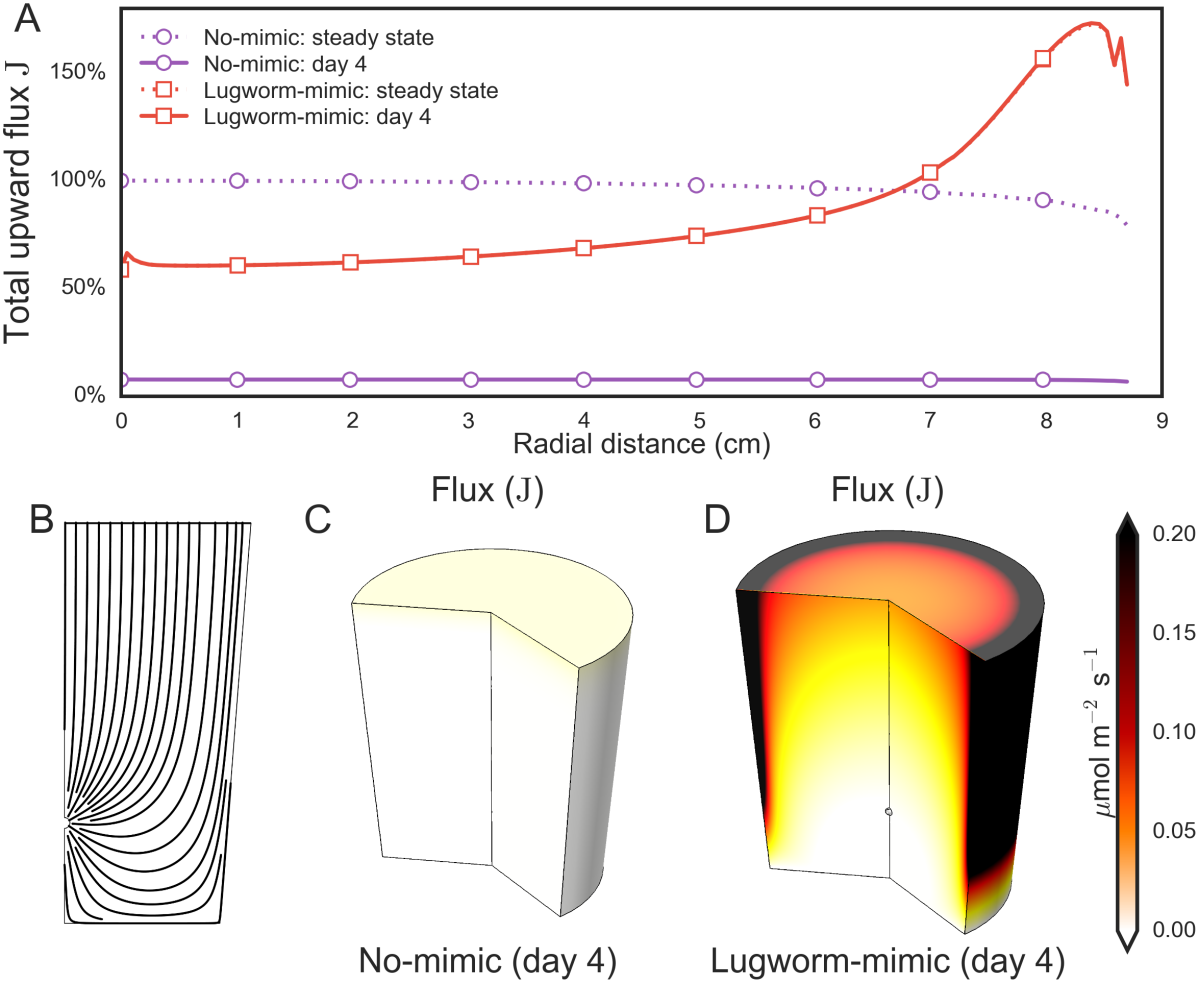
Results of modeling the total upward flux of nutrients through the sediment—water interface in the experimental containers with and without a lugworm mimic. (A) Radial distributions of the total upward flux **J** at the end of the incubation experiment and at a projected steady-state. The plotted flux is normalized to the steady-state value in the no-mimic treatment at the center of the container. The distribution for the lugworm-mimic treatment reached a steady-state before day 4. (B) Modeled streamlines of the porewater flow induced by the lugworm mimic in the experimental container. (C-D) Modeled distributions of the total upward flux of nutrients at the end of the incubation in the experimental containers with and without a lugworm mimic.

To interpret the observed MPB growth patterns in the lugworm-mimic experiment, we calculated for both treatments the total upward nutrient flux (**J**) across the sediment–water interface as a temporal evolution towards an eventual steady-state. We assumed that the nutrient generation rate inside the sediment was spatially uniform and the same for both treatments with and without the lugworm-mimic. Firstly, the model revealed that the radial profile of **J** was largely flat throughout the temporal evolution for the no-mimic treatment, whereas it displayed a marked increase towards the container's edge for the lugworm-mimic treatment (Fig 4A), similar to the profile of MPB growth in the lugworm-mimic experiment (compare with Fig 3D). The radial increase of the surface flux corresponds with the increasing path-length of the flow lines of porewater in the lugworm-mimic treatment (Fig 4B). Secondly, the distribution of **J** in the lugworm-mimic treatment reached steady-state after two days whereas the no-mimic treatment required >1000 days. This implies that during the four days of incubation in our lugworm-mimic experiment (Fig 3) the upward flux of nutrients in the lugworm-mimic containers most likely reached a steady-state whereas it remained very low (<5% of maximum potential) in the no-mimic containers (Fig 4A, 4C and 4D). It is important to note that under steady-state conditions, which were clearly not reached in the experimental containers without lugworms mimics, the nutrient flux integrated over the entire sediment–water interface in the containers would be the same for both treatments, although their spatial distributions would differ considerably. This follows from the fact that the rates of nutrient generation within the sediment volume were assumed to be the same in both modeled treatments, and only the dominant transport mode differed.

### In-situ MPB distribution in the presence and absence of lugworms

**Fig 5:**
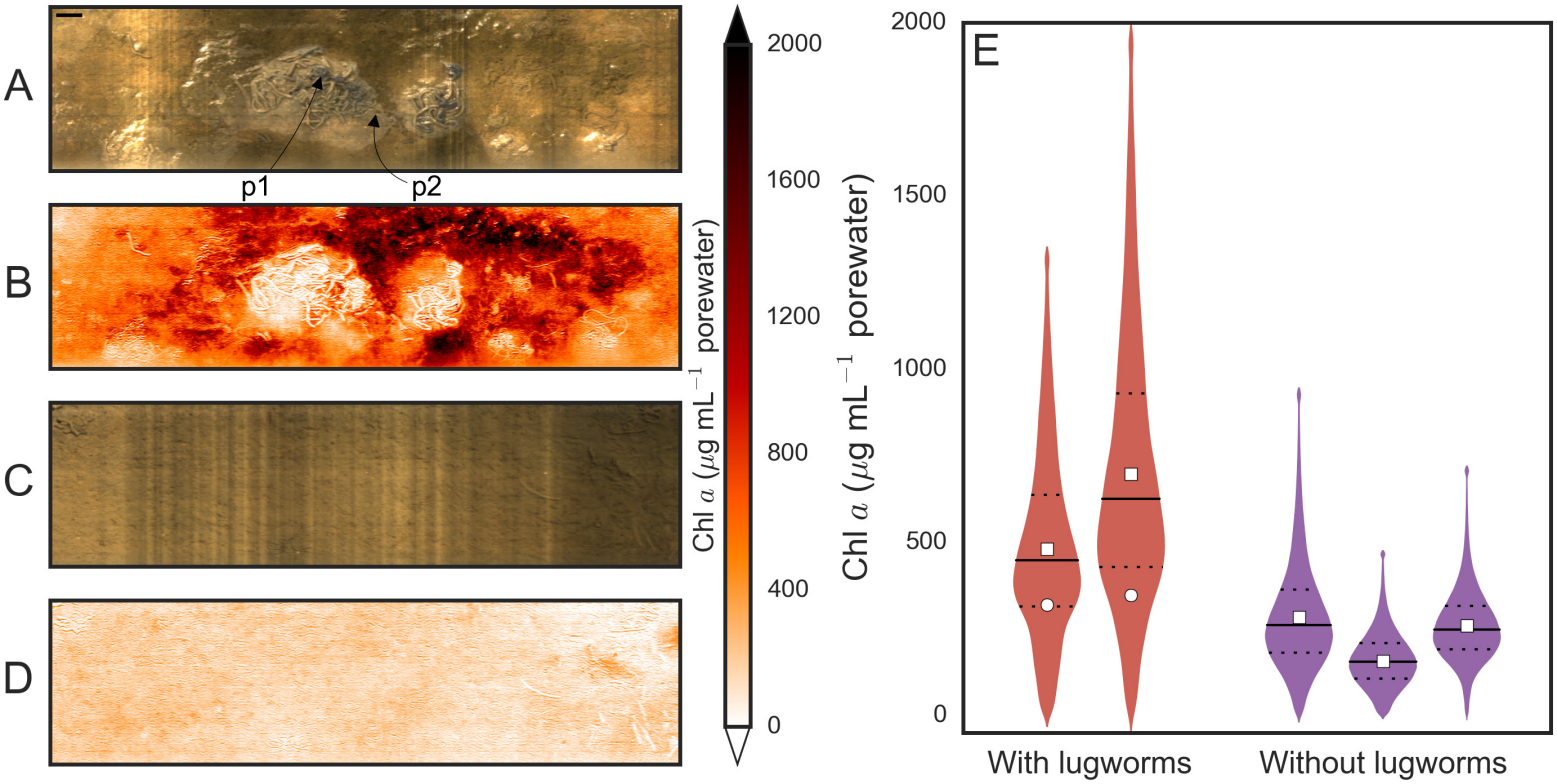
Chlorophyll *a* maps of in-situ experimental plots with and without lugworms. (A–D) Examples of maps at the surface of permeable intertidal sediments with (A, B) and without (C, D) lugworms (scale bar 2cm). The true-color images of the sediment (A,C) are shown together with the corresponding chlorophyll *a* maps (B,D) of the sediment surface. The true-color image A shows biogenic structures of lugworms, such as fecal mounds (p1) and feeding funnels (p2). (E) The distribution of surficial chl *a* concentrations of replicate plots as violin plots, with the inner horizontal lines indicating the quartile levels and the square markers the average values. For the lugworm plots, sediment areas that were affected by reworking were averaged separately (circle markers).

The true-color and chl *a* maps from the in-situ experiment showed a clear difference between the experimental plots with and without lugworms after five weeks of incubation (Fig 5A–5D). The presence of lugworms was manifested by biogenic structures such as fecal mounds or feeding funnels visible at the sediment surface (p1 and p2 in Fig 5A). Similar to results obtained in laboratory containers (compare Figs 1A and 5B), freshly defecated sediment was characterized by very low chl *a* concentrations, while sediment that was not visibly affected by reworking (sediment excluding feeding funnels and fecal mounds) showed greatly enhanced chl *a* content, indicating an increased MPB population in these regions.

As shown by the violin plots summarizing the pixel statistics of each replicate experimental plot (Fig 5E), the overall surficial MPB stock, measured as the mean chl *a* over the scanned area, was about 250% higher (F = 13.94, p = 0.034) in the sediment with lugworms than in the lugworm-free sediment. The spatial heterogeneity of the surficial MPB stock, measured as the standard deviation over the scanned area, in the sediment with lugworms was 260% higher (F = 17.14, p = 0.026) than in the lugworm-free sediment. Based on the true-color images of the sediment surface, we sectioned the maps of the sediment with lugworms into regions affected by reworking and those that were visibly undisturbed. This sectioning revealed that the sediment regions undisturbed by reworking contained on average 330% higher chl *a* concentration than the sediment without lugworms, whereas chl *a* concentration in the reworked sediment was on average only 140% higher.

## Discussion

### Role of lugworm bioturbation on surficial MPB biomass

Lugworms are a classic example of bioturbating ecosystem engineers [32]. The aim of this study was to quantify the effects of their two main types of activity—sediment reworking and burrow ventilation—on the small-scale dynamics of the surficial MPB biomass. On the one hand, sediment reworking by lugworms decreases the surficial MPB biomass through the combination of feeding activity (subduction of the sediment–water interface and digestion during gut passage) and burial (defecation on sediment surface). On the other hand, we hypothesized that the advective porewater transport induced by burrow ventilation [13, 15] enhances the nutrient flux from deeper sediment layers and thus promotes MPB growth at the sediment surface.

Our results show that both of these phenomena are important drivers of MPB biomass distribution in the studied sediments. Specifically, the fecal casts and feeding funnels had markedly depleted concentrations of surficial MPB biomass, while the biomass in the surrounding sediment areas was clearly elevated in comparison to the sediments without lugworms (Figs 1 and 5). Overall, the combined effect observed over the time-scale of days to weeks was a 150%–250% higher mean and 280% larger variability of the surficial biomass in the sediments with lugworms as compared to the sediments without lugworms (Figs 2 and 5; S1 Table).

In nature, the surficial MPB stock can possibly be affected by several other factors in addition to lugworm bioturbation. For example, tidal currents cause surficial erosion, alter rates of recolonization of depleted area such as fecal castings, and cause porewater advection in surface sediment layers which may deplete nutrients. However, when we eliminated these additional factors and incubated single lugworm individuals in a constrained sediment volume under controlled laboratory conditions, we obtained very similar results to those from the field. This shows that bioturbation is a factor that imposes significant control over the dynamics of surficial MPB biomass in natural permeable sediments on the scale of days to weeks.

A specific aim of our experiments was to study the role of bioadvection induced by lugworms in the dynamics of surficial MPB biomass. To do this, we replaced real lugworms with a mechanical device (“robolug”) that realistically mimics pumping of water into sediments associated with burrow ventilation by lugworms. After four days, we observed a clear increase in the surficial MPB biomass at the edge of the experimental containers with the lugworm-mimics as compared to containers without the mimic (Fig 3). Furthermore, the relative increase was similar to the laboratory containers with and without real lugworms after 11 days (compare Figs 2 and 3). This shows that in sand-flats with hydraulic ecosystem engineers such as lugworms, bioadvection is a significant driver of MPB growth.

To understand the mechanism behind this MPB growth stimulation, we modeled transport of a growth-limiting nutrient (e.g., NH_4_^+^) through the sediment in the same geometry and over the same time-scale as in the lugworm-mimic experiment assuming that this nutrient is generated in the bulk sediment via organic matter remineralization. First, it should be noted that the assumption of a uniform and constant rate of nutrient remineralization in our model is a simplification of the processes occurring in natural sediments [33]. While it is likely a reasonable simplification for the short duration of our experiments, natural sediments will not reach the 'predicted' steady-states (e.g., Fig 4A) as nutrient remineralization in sediments is affected by additional factors that were not included in the model, such as organic matter lability [34], bioturbation [35] and redox conditions [36, 37], and is ultimately constrained by the bio-availability of organic matter within the sediment. Nevertheless, the modeling results showed that the nutrient supply towards the sediment–water interface during the incubation period of 4 days was likely far greater when the transport by bioadvection was active then when it was governed only by diffusion (Fig 4A). Furthermore, the predicted radial profile of the nutrient flux at the sediment–water interface was similar to that of the net increase in the surficial MPB biomass observed during the lugworm-mimic experiment (Fig 3A). This implies that the most likely mechanism behind stimulation of MPB growth in sediments affected by bioadvection is the enhanced transport of remineralized nutrients from deeper sediment layers towards the sediment surface.

It should be noted that we did not measure nutrient fluxes in our experiments. However, enhanced rates of organic mineralization [33, 35] and solute exchange across the sediment–water interface in bioirrigated sediments, including oxygen fluxes into [13, 38], and CO_2_ fluxes out of the sediment [39, 40] are well documented. Also elevated fluxes of NH_4_^+^ from bioirrigated sediments have been reported [41], although they can sometimes be converted into elevated fluxes of N_2_ by coupled nitrification-denitrification. We can be therefore confident about our conclusion that the elevated nutrient flux driven by porewater advection was responsible for the enhanced growth of MPB observed at the surface of sediments with lugworms.

Interestingly, Na et al. [42] reported a very high initial release of NH_4_^+^ following the introduction of lugworms to sediment containers, which was attributed to the flushing of accumulated diagenetic products out of the sediment cores. This initial flushing of accumulated products is likely relevant to our experiments as it is possible that the sediment homogenization process introduced electron-rich acceptors (e.g., O_2_) in the porewater which may have resulted in a high initial diffusive flux of mineralization products in both treatments. This might explain the initial growth of MPB observed in both containers with and without lugworms (Fig 2), followed by divergent responses in the treatments. To some extent, this may also be a relevant feature in the field as lugworms are known to relocate from time to time [43].

#### Implications

While sediments have generally been viewed as an unlimited source of nutrients for microphytobenthos, nutrient supply to the surface may actually become limiting under diffusive conditions, especially during daytime when MPB activity and nutrient demand are maximal [44–46]. In these situations bioadvective upwelling of porewater nutrients from deeper sediment may be an important mechanism to alleviate nutrient limitation on MPB growth.

There has been considerable discussion about the 'advective footprint' of an individual lugworm in permeable sediments with respect the size of the sediment area affected by solute transport, with values ranging from 5 to 30 cm^2^ [14, 31, 47, 48]. These predictions, mostly based on modeling, were challenged by field measurments of the spatial extent of porewater pressure dynamics in the presence of lugworms [15]. Although model simulations based on these measurements did confirm a complete porewater turnover below an area of 30 cm^2^, they also suggested a significant impact of a single lugworm over much larger areas (e.g. 10% of the porewater is replaced every day by each lugworm below an area of >450cm^2^; [15]). Our experiments support this, as demonstrated by the size of the surficial sediments where MPB growth was significantly enhanced through biologically or mechanically induced porewater advection (180–350 cm^2^; Figs 1 and 3). Since densities of hydraulically active infaunal organisms are often large enough that the individual hydraulic footprints overlap, the entire sediment surface should then be affected by bioadvection [15, 32]. Thus, 'community gardening' may be effective not only in the immediate vicinity of the locations of water injection but likely involves the entire sediment surface of densely populated sites that can cover hundreds of square kilometers, as in the case of the lugworm-inhabited sediments of the Wadden Sea [19].

It should be noted that the increased MPB growth in bioirrigated sediments that we have consistently observed in our laboratory experiments does not necessarily translate into increased MPB standing stocks in the field, e.g., as observed in our small-scale field experiment (Fig 5). Long-term and large-scale experimental exclusion of lugworms resulted in increased chl *a* standing stock in the surficial sediments [38], while chl *a* standing stocks were not affected in a lugworm addition field experiment [49]. We argue that estimates of MPB standing stocks are likely affected by feeding rates and hydrodynamic conditions in the field. This also emphasizes that MPB standing stocks must be interpreted with caution as they may not necessarily reflect the productivity of the system.

Overall, we demonstrate that bioadvective porewater transport induced by hydraulically active infauna, such as lugworms, is a relevant mechanism that significantly shapes the distribution of MPB in permeable marine sediments, and should be considered in analyses of benthic ecology of permeable sediments within the photic zone. The fertilizing effect of bioadvective nutrient supply is likely critical in supporting primary productivity rates that are sufficiently high to sustain deposit-feeding populations in organic poor sediments.

## Supporting Information

S1 Table Statistical results on differences in treatments. Statistical testing of the difference in the surficial chl a distribution in sediment containers with and without lugworms. The mean and standard deviation were tested separately.
(DOC)
