# Supplementary material for: Effects of Bioadvection by Arenicola marina on Microphytobenthos in Permeable Sediments
Source: PLoS One. 2015 Jul 31;10(7):e0134236. doi: 10.1371/journal.pone.0134236 (PMC4521690; doi:10.1371/journal.pone.0134236)
Supplement: S1 Table — Statistical testing of the difference in the surficial chl a distribution in sediment containers with and without lugworms. The mean and standard deviation were tested separately. (DOC) [file pone.0134236.s001.doc]

**S1 Table. Statistical results on differences in treatments.**

Statistical testing of the difference in the surficial chl a distribution in sediment containers with and without lugworms. The surficial chl *a* mean and standard deviation were tested separately.

|  | Timepoint | Treatment | Replicates | Mean value | Kruskal-Wallis |
| --- | --- | --- | --- | --- | --- |
| Surficial chl *a* mean  (µg chl *a* ml^-1^PW) | day 1 | with worm | 3 | 594.9 ± 42.8 | F: 0.05  p: 0.83 |
|  |  | no worm | 3 | 600.9 ± 36.4 |  |
|  | day 4 | with worm | 3 | 1016.8 ± 108.6 | F: 0.43  p: 0.51 |
|  |  | no worm | 3 | 991.7 ± 55.5 |  |
|  | day 11 | with worm | 3 | 1211.7 ± 273.0 | F: 3.86  p: 0.05 |
|  |  | no worm | 3 | 786.0 ± 51.8 |  |
| Surficial chl *a* standard deviation  (µg chl *a* ml^-1^PW) | day 1 | with worm | 3 | 128.6 ± 17.1 | F: 2.33  p: 0.13 |
|  |  | no worm | 3 | 117.5 ± 3.9 |  |
|  | day 4 | with worm | 3 | 254.3 ± 59.8 | F: 3.86  p: 0.05 |
|  |  | no worm | 3 | 154.9 ± 0.4 |  |
|  | day 11 | with worm | 3 | 490.8 ± 54.7 | F: 3.86  p: 0.05 |
|  |  | no worm | 3 | 177.6 ± 33.0 |  |
